# Analysis of Small RNAs of Barley Genotypes Associated with Resistance to Barley Yellow Dwarf Virus

**DOI:** 10.3390/plants9010060

**Published:** 2020-01-02

**Authors:** Jana Jarošová, Khushwant Singh, Jana Chrpová, Jiban Kumar Kundu

**Affiliations:** Plant Virus and Vector Interactions, Crop Research Institute, Drnovská 507, 16106 Prague, Czech Republic; j.jarosova@vurv.cz (J.J.); singh@vurv.cz (K.S.); chrpova@vurv.cz (J.C.)

**Keywords:** BYDV, NGS, sequencing, sRNA, miRNA, *Ryd*2

## Abstract

Barley yellow dwarf virus (BYDV) causes an often-devastating disease of cereals that is most effectively controlled by using plant genotypes that are resistant or tolerant to the virus. New barley lines Vir8:3 and Vir13:8, with pyramided resistance genes against different pathogens and resistance gene *Ryd*2 against BYDV, are currently being tested. Because microRNAs (miRNAs) are associated with antiviral plant defense, here we compared the miRNA profiles in these lines and in cultivar Wysor (carrying one resistance gene, *Ryd*2), with and without BYDV infection and after feeding by virus-free aphids, to determine whether the miRNA profile in the resistant variety bear similarities with the newly developed lines. The BYDV titer for each group was also determined and compared to the titer in sensitive cultivar Graciosa. Among 746 miRNAs identified in barley, 66 were known miRNAs, and 680 were novel. The expression of 73 miRNAs differed significantly after BYDV infection, including the strong, specific upregulation of novel miRNA10778 that was conserved across all the barley genotypes. This miRNA belongs to the H box and ACA box (H/ACA) snoR14 family of RNAs (Rf01280) and is associated with pseudourydilation. The expression of 48 miRNAs also differed depending on the barley genotype. The profile of miRNAs expressed in Vir8:3 and Vir13:8 in response to BYDV was similar and differed from that of Wysor. Insights into the expression patterns of miRNAs in response to BYDV in barley provided here will benefit further studies toward understanding the resistance mechanisms and developing novel strategies against virus infections.

## 1. Introduction

Barley yellow dwarf viruses (BYDV), a group of viruses assigned to the genus *Luteovirus* (BYDV-kerII, BYDV-kerIII, BYDV-MAV, BYDV-PAS, and BYDV-PAV) [1], are aphid-transmitted viruses that infect cereals worldwide, including wheat, barley, oats, maize, and rice [2]. Yield losses of up to 80% have been reported [3], but the loss varies greatly depending on the virus isolate, cultivar, time of infection, and environment [4]. The most effective and sustainable control method to date is the use of plant material that is resistant or tolerant to the virus complex [5], but cereal resistance to BYDV is rare and complicated.

Despite considerable work to develop resistant cultivars and lines, the results have been mixed [6]. However, a European program to pyramid resistance genes was successful in registering the Italian feed barley cultivar Doria, which carries resistance genes effective against BYDV (*Ryd*2), Barley yellow mosaic virus (BaYMV1) and Barley mild mosaic virus (BaMMV) (*rym*4), and *Pyrenophora graminea* (*Rdg*1) [7]. In addition, crosses between Doria and winter six-rowed, non-malting barley cultivar Traminer, which carries resistance to *Blumeria graminis* and *Puccinia hordei*, have resulted in some promising lines. Two of these lines, Vir8:3 and Vir13:8, were tested in the present study. Yield loss from these two lines is low when they are infected with BYDV, which are comparable to the yield losses for the resistant variety Wysor.

Analysis of the RNA transcriptome of plant genotypes is important for understanding differential gene expression in resistant and susceptible host plants during their interactions with a pathogen. RNA sequencing (RNA-Seq) can provide high-throughput data for both host and virus transcriptomes [8] to profile gene expression during infection. Of particular interest are virus-derived small RNAs because of their potential function as regulators of the host transcriptome machinery [9,10] for the antiviral defense and counter-defense strategy in plant species known as RNA silencing [11].

RNA silencing is triggered by double-stranded (ds) RNA, which serves as a substrate for Dicer-like ribonucleases (DCL) to produce two major classes of small RNAs (sRNAs): small RNAs derived from single-stranded precursors with a hairpin structure (hpRNAs) and those derived from dsRNA precursors (small interfering RNAs (siRNAs)). MicroRNAs (miRNAs) belong to the hpRNAs [12]. The distinction between miRNAs and siRNAs in their functionality and mode of operation is less pronounced [13,14]. sRNAs play important roles in the developmental regulation and environmental adaptation of plants. miRNAs control the expression of many key regulatory genes by binding messenger RNA (mRNA), either targeting destruction of the mRNA by cleavage or preventing its translation into protein [14,15]. Many miRNAs are associated with host responses to virus infection [16], including host resistance to viral infection [17,18], such as miR6019 and miR6020 in tobacco to tobacco mosaic virus (TMV) [19]; miR160, miR166, miR167, miR171, and miR396 in rice to rice stripe virus (RSV) [20]; and miR156 in tomato to tomato leaf curl virus (ToLCV) [21].

In the present study, we used sRNA-Seq and RT-qPCR to analyze the small RNA profile in the new barley lines Vir8:3 and Vir13:8 and in the BYDV-resistant cultivar Wysor (carrier of *Ryd*2). Vir8:3 and Vir13:8 phenotypically have a similar level of resistance to BYDV in field and greenhouse experiments (unpublished data). We compared the small RNA sequences in the three lines to better understand defense-related miRNA expression against virus infection in barley plants and to see whether the miRNAs’ profiles in the resistant lines bore similarities with the newly developed lines.

## 2. Material and Methods

### 2.1. Experimental Conditions

**Figure 1:**
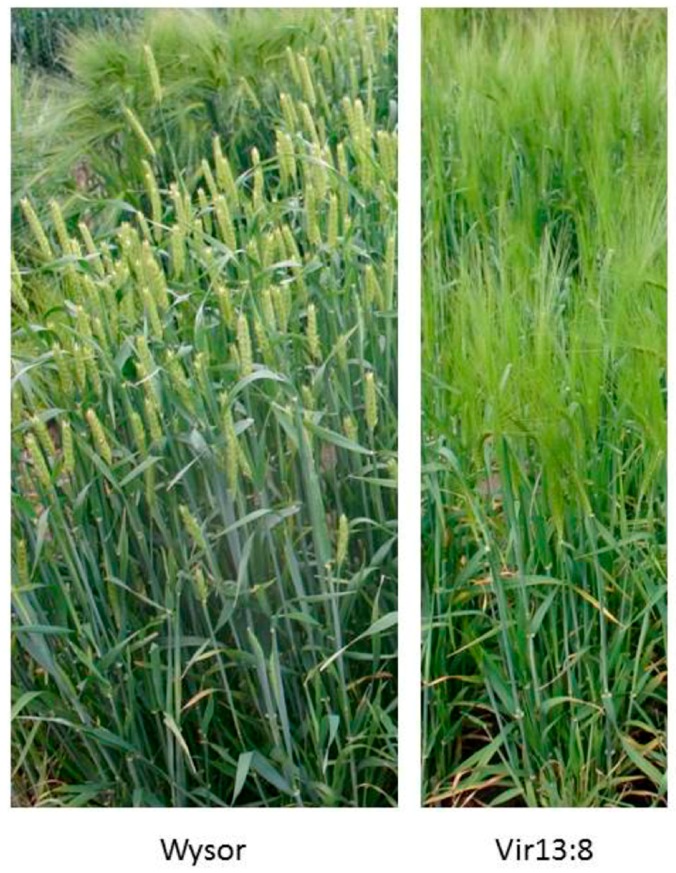
Cultivars used in the present study. Wysor has *Ryd2* for moderate resistance to BYDV, whereas line Vir13:8 is a cross between six-rowed, non-malting winter barley cultivar Tramine and the *Ryd2*-carrying, two-rowed feed barley cultivar Doria.

Four genotypes of barley (*Hordeum vulgare*) were selected: Graciosa, as a susceptible cultivar (and used only for the qPCR to quantify the viral titer as a control); Wysor carrying *Ryd*2, for its moderate resistance to BYDV; and lines Vir8:3 and Vir13:8, as crosses between six-rowed, non-malting winter barley cultivar Tramine and the *Ryd*2-carrying, two-rowed feed barley cultivar Doria (Figure 1).

The aphids (*Rhopalospiphum padi*) used for the experiment were reproduced parthenogenetically from one mother. Before the experiment, half were kept on BYDV-free barley plants; the second half were kept for a week on BYDV-PAV-infected barley plants to acquire the virus.

Barley seeds were planted in 10 × 10 cm plastic pots filled with a pre-mixed sterilized substrate, one plant per pot. The plants were inoculated at the age of 14 days with BYDV-PAV from the Virus Collection of the Crop Research Institute, Prague [22], using viruliferous aphids. The aphids were then allowed to feed on 2-week-old plants for 3 days, after which, plants in all groups were treated with one dose of the insecticide Acetamiprid (Mospilan 20 SP, Sumi Agro, Prague, Czech Republic) according to the manufacturer’s recommendations. The experiment was carried out in controlled conditions (21 °C, 16-h light, 60% humidity) in a greenhouse in separate insect-proof net cages with three treatment groups: control healthy plants (hereafter, healthy group); plants on which BYDV-free aphids were fed (aphid group); and plants on which aphids carrying BYDV-PAV fed (BYDV group). Triplicates were used for each genotype and each treatment. The samples were collected at 7 and 14 days post inoculation (dpi); therefore, three samples were collected for each genotype, treatment, and time point. Samples were snap-frozen, ground in liquid nitrogen, and stored in 100 mg aliquots at −80 °C. The 7-dpi samples were used only for qPCR quantification of the BYDV titer; the 14-dpi samples were used for both determining the BYDV titer and deep sequencing. Twenty-seven samples in total were used for deep sequencing and 72 samples were used for RT-qPCR analysis. The samples were not pooled, but rather analyzed individually.

### 2.2. RNA Isolation

Total RNA was isolated with a combination of Trizol-based reagent RNA blue (Top-Bio, Vestec, Czech Republic), column-based system Quick RNA Plus Kit (Zymo Research, Irvine, CA, USA), and Wash buffer 1 from the column-based system Spectrum Plant Total RNA Kit (Sigma Aldrich, St. Louis, MO, USA). DNase treatment was carried out in a column. For confirming the expression of miRNAs using qPCR, small RNAs were captured from the total RNA using a Plant microRNA Purification Kit (Norgen, Thorold, ON, Canada) according to the kit protocol. RNA integrity was confirmed using 1.0% agarose gel electrophoresis, and the total RNA was quantified with a NanoDrop 1000 spectrophotometer (Thermo Scientific, Waltham, MA, USA) and a Quantus Fluorometer (Promega, Madison, WI, USA).

### 2.3. qPCR Conditions

For quantifying the BYDV titer, cDNA was synthesized using 1 µg of total RNA, random hexamers, and a RevertAid First Strand cDNA Synthesis Kit (Thermo Scientific) according to the manufacturer’s instructions. The cDNA was diluted 10-fold for the qPCR assays in a LightCycler 480 (Roche, Basel, Switzerland) using LightCycler 480 SYBR Green I Master (Roche), according to the methods of Jarošová and Kundu [23]. For confirming the expression of barley miRNAs, cDNA was synthesized using 500 ng of RNA, specific stem-loop primers, and a RevertAid First Strand cDNA Synthesis Kit (Thermo Scientific). The assay was carried out according to [24] and multiplexed as described by Turner [25] (four targeted cDNAs in one reaction). For the normalization strategy adapted from Ferdous [26] three barley genes were selected (*snoR14*, *snoR23*, and *hvu-MiR168*), and the normalized expression was calculated as an average expression. The primers for cDNA synthesis and for qPCR are given in Supplementary File S1. The relative expression of all miRNAs was calculated using the 2^−ΔΔCt^ method [27].

### 2.4. Library Preparation and sRNA-Seq

After ribosomal RNA depletion (RiboMinus Plant Kit for sRNA-Seq; Invitrogen, Waltham, MA, USA), NEBNext Small RNA Library Prep for Illumina (New England Biolabs, Ipswich, MA, USA) was used according to the manufacturer’s instructions for Next generation sequencing (NGS) library preparation. For the library quantifications and quality controls, a Bioanalyzer High Sensitivity DNA Kit (Agilent, Santa Clara, CA, USA) was used. The sequencing run was carried out using HiSeq4000 (Illumina, San Diego, CA, USA). In total, 391,088,557 single-end 50-bp reads with a high confidence were generated.

### 2.5. Bioinformatic Analyses of sRNA-Seq Data

The quality of raw reads was evaluated using FastQC (v0.11.5) (Babraham Institute, Cambridge, UK) (https://www.bioinformatics.babraham.ac.uk/projects/fastqc/) and MultiQC (1.0.dev0) [28] (https://multiqc.info/). Adaptor sequences were removed using cutadapt (v1.9.1) [29] (https://cutadapt.readthedocs.io/en/stable/). The trimmed reads were aligned to the noncoding RNA (ncRNA) and chloroplast reference using Torrent Mapping Alignment Program (TMAP, v.5.10.11) (https://github.com/iontorrent/TMAP). Unmapped reads (adjusted reads) were aligned to the *Hordeum vulgare* microRNA database (miRBase v22.0) [30], and reads that were not mapped to the reference were used as input for the miRPlant tool (v.6) [31] to predict novel miRNAs. The predicted miRNAs with a score lower than 4 and not located in chromosomes 1 to 7H were filtered out. The novel miRNAs were divided into RNA families based on the Rfam database (v.11) (https://rfam.xfam.org/). The adjusted reads were aligned to novel and known miRNA reference sequences using Tmap. The read groups were replaced in Binary Alignment Map (BAM) files using PicardTools (v2.4.1) (https://broadinstitute.github.io/picard/). The number of reads mapped to the *Hordeum vulgare* miRNA and novel reference miRNA was extracted and used for differential gene expression analysis. Differential gene expression was analyzed using the R bioconductor packages DESEq2 [32] and EdgeR [33]. The miRNAs with log fold-change (Log_2_FC) of more than 1.5 (1.2) or less than Log_2_FC—1.5 (1.2) and with *p*-value less than 0.05 were considered significantly differentially expressed. Gene ontology analysis was performed using the g:Profile (g:GOSt) web server (https://biit.cs.ut.ee/gprofiler/gost). Functional enrichment was assessed using *Hordeum vulgare* and the default parameters. The significant threshold was selected using g:SCS and the significant results with *p*-value < 0.05 were further processed for the analysis. Domain architecture analysis was performed using the InterPro protein sequence analysis and classification database (https://www.ebi.ac.uk/interpro/).

### 2.6. siRNA Analysis

For the siRNAs analysis, reads less than 15 nucleotides (nt) or longer than 30 nucleotides were trimmed out using cutadapt (v1.9.1) [29]. Quality control of siRNA reads was checked using FastQC (https://www.bioinformatics.babraham.ac.uk/projects/fastqc/). The high-quality reads were aligned with the *Hordeum vulgare* genome from EnsemblPlants (International Barley Genome Sequencing Consortium) using Bowtie version 1.2.2, allowing up to three mismatches (http://bowtie-bio.sourceforge.net/index.shtml). Unaligned reads were mapped to the BYDV-PAV genome (National Center for Biotechnology Information accession D85783) using Bowtie version 1.2.2.

### 2.7. Statistical Analyses

General statistical analyses (ANOVA, Pearson correlation coefficients) were done using GraphPad Prism 8 (GraphPad Software, San Diego, CA, USA). The normality of the distribution of the data was tested using the D’Agostino and Pearson normality test, the Shapiro–Wilk normality test, and the Kolmogorov–Smirnov normality test. The experimental data were analyzed using either Kruskal–Wallis ANOVA or two-way ANOVA, followed by the Bonferroni post hoc tests, where appropriate.

## 3. Results

### 3.1. qPCR Analyses of BYDV Titer

**Figure 2:**
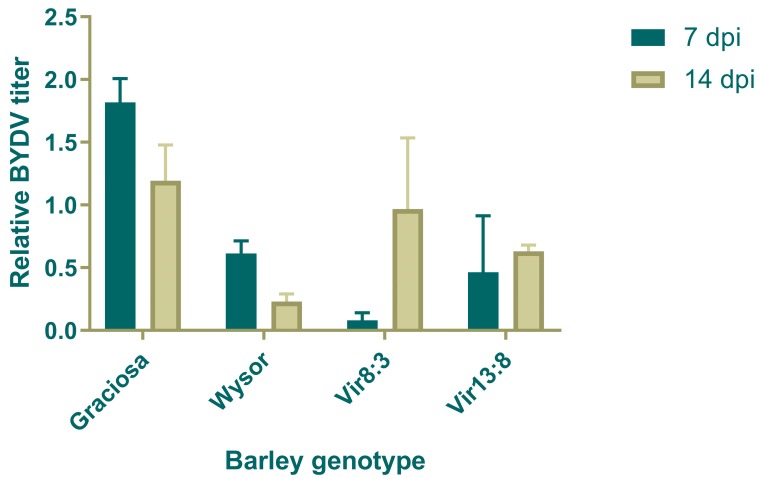
Relative Barley yellow dwarf virus (BYDV) titer in infected barley (*Hordeum vulgare* L.) genotypes 7 and 14 days post inoculation (dpi). The standard deviation is expressed as error bars. The whole plants (above-ground biomass) was collected and assayed. Relative titer values were calculated using the 2^−ΔΔCt^ method.

When the BYDV titer was determined at 7 and 14 dpi, BYDV was detected in all aphid-inoculated plants, and control plants were virus-free. The highest titer was reached in the susceptible control cultivar Graciosa and was approximately three times higher than in Wysor (*Ryd*2 carrier) at 7 dpi and five times higher at 14 dpi. The titer in both Graciosa and Wysor was higher at 7 dpi than at 14 dpi, but the fall in titer in Wysor was sharper than in Graciosa (Figure 2). The BYDV titer in lines Vir8:3 and Vir13:8 was (with one exception) always lower than in Graciosa, and at 7 dpi was similar or lower than in Wysor. On the other hand, at 14 dpi, the BYDV titer of Wysor was 2–3 times lower than in the tested lines. In these lines, the tendency was the opposite; the BYDV titer increased twofold to fivefold from 7 to 14 dpi.

### 3.2. Small RNA Deep Sequencing

For the three genotypes and three treatments (healthy controls, “aphid” plants on which virus-free aphids were allowed to feed, and BYDV-infected plants), each with three replications, 27 small RNA libraries were generated. The sequencing run on HiSeq4000 generated 391,088,557 single-end 50-bp reads with a high confidence. The results were stored in 27 separate files, each containing from 11.8 to 19.9 million reads (average of 14.5 million reads per file; Supplementary File S2). Raw data were automatically processed using the Basespace cloud interface (https://support.illumina.com/help/BaseSpace_Sequence_Hub/Source/Informatics/BS/Apps_swBS.htm) with default settings, and base calling, adapter clipping, and quality filtering were carried out. Reads less than 15 bp were filtered out. Trimmed reads were aligned to the ncRNA and chloroplast reference sequences, and only unmapped reads (50–63% of total raw reads) were extracted.

**Figure 3:**
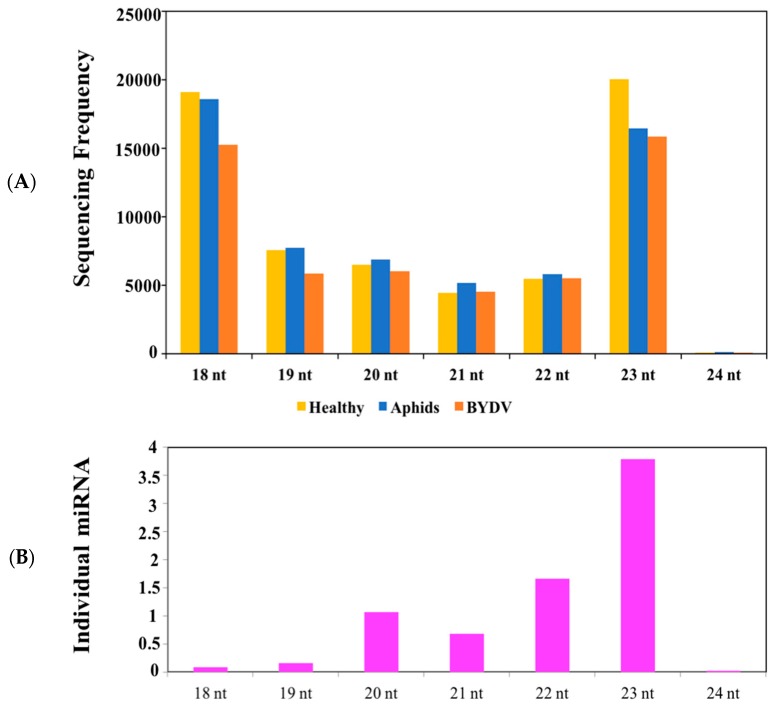
Length distribution of individual miRNAs. (**A**) Total calculated reads of all miRNAs from healthy, aphid-infested non-infected, and BYDV-infected plants. (**B**) Individual miRNAs.

The length of novel miRNAs ranged from 18 to 23 nt; 18-nt and 23-nt miRNAs were the most abundant in terms of total sequencing readings (Figure 3). The total miRNAs sequence reads decreased slightly among the treatment groups (highest to lowest abundance): healthy (6,324,030 reads) > aphid (6,083,004 reads) > BYDV (5,320,467 reads) (Supplementary File S3). The same pattern was observed among individual genotypes: line Vir8:3 (6,553,664 reads) > line Vir13:8 (6,121,019 reads) > cultivar Wysor (5,052,818). When individual triplicates were compared, the healthy > aphid > BYDV trend was present for Vir13:8 and Wysor, but not for line Vir8:3 (Table 1).

For individual miRNAs, the most abundant lengths were 23 nt (55% of all miRNAs), 22 nt (22%), 20 nt (15%), and 21 nt (5%). The shortest miRNAs (19 nt, 2%; 18 nt, 1%) were the least abundant (Figure 3).

### 3.3. Identification of Known and Novel miRNAs in Barley

Alignment of the adjusted reads for the barley small RNAs against known reference miRNAs in *Hordeum vulgare* allowed us to identify 66 known miRNAs belonging to 12 RNA families (Supplementary File S3). The abundance of hvu-MiR5053, hvu-MiR6178, hvu-MiR6179, hvu-MiR6186, hvu-MiR6187, hvu-MiR6202, hvu-MiR6206, hvu-MiR6207, hvu-MiR6210, and hvu-MiR6211 was low in all sRNA libraries (Supplementary File S3). Highly abundant in all samples were hvu-MiR156b, hvu-MiR159a, hvu-MiR166c, and hvu-MiR444a.

**Figure 4:**
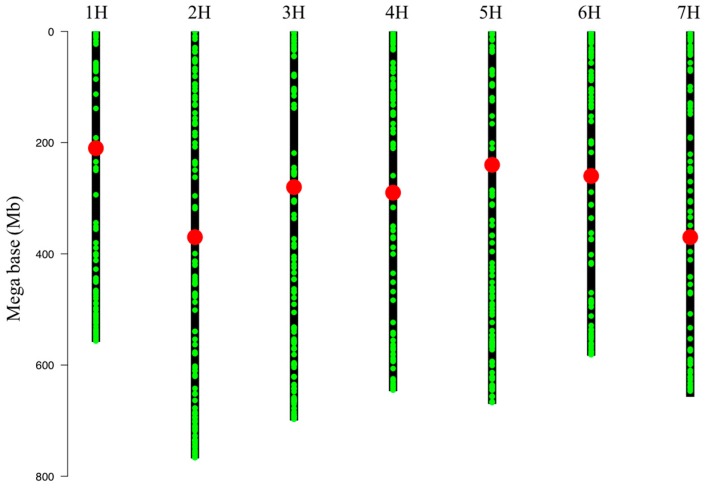
Karyogram showing the chromosomal positions of novel miRNAs on chromosomes 1 to 7H of barley (*Hordeum vulgare*). The position of each miRNA is shown using a green circle. Centromeres are represented using a red oval. The final karyogram was drawn using the ggplot function in the ggplot package of R and an R script, as described [34].

When unmapped reads were used to predict novel miRNAs using plant miRNA (miRPlant), nearly 40,000 new miRNA sequences were predicted. We selected 680 of the high quality predicted novel miRNAs for further study and named them using the format novelMiR plus number (they will be given their final names and deposited in the miRBase.org upon acceptance of this manuscript). Their predicted locations on one of the seven specific chromosomes (1–7H) are given in Figure 4. Among the 680 novel miRNAs, 662 miRNAs were grouped into 161 RNA families based on the Rfam database (Table 2; Supplementary File S4).

The most abundant novel miRNAs were novelMiRNA_37561, novelMiRNA_37081, novelMiRNA_34259 (hundreds of thousands of reads in each sample), followed by novelMiRNA_3949, novelMiRNA_35669, novelMiRNA_35061, novelMiRNA_24768, novelMiRNA_24765, novelMiRNA_23437, novelMiRNA_27733, novelMiRNA_14164, and novelMiRNA_14050 (tens of thousands of reads in each sample). The least abundant were novelMiRNA_7314, novelMiRNA_7315, novelMiRNA_10399, novelMiRNA_24421, novelMiRNA_31433, novelMiRNA_7317, novelMiRNA_15682, novelMiRNA_5298, novelMiRNA_2323, novelMiRNA_10964, novelMiRNA_5163, and novelMiRNA_13760 (10–20 reads in each sample).

The sequences of novel miRNAs and known miRNAs were concatenated into a new reference sequence. The adjusted reads were aligned against this new miRNA reference sequence, and variants were called using FreeBayes software [35]. In total, 12,151 variant sites were detected (of which 9656 were biallelic and 2495 where multiallelic). The number of adjusted and mapped reads to each miRNA was extracted and used for differential expression analysis using two approaches (DESeq2 and EdgeR). Five groups (A–E) and several subgroups were determined based on the experimental conditions (see Supplementary File S5). To analyze differential changes in the expression of miRNAs among barley types and treatments after inoculation, we calculated the relative expression level of the miRNAs in individual groups using the log fold-change (Log_2_ FC). The miRNAs with a log fold-change more than 1.5 or less than −1.5 and with *p*-values lower than 0.05 were considered significantly differentially expressed, except in group A comparing virus-free aphid treated and BYDV samples, where no result was found with these settings, and therefore the log fold-change threshold was set to 1.2 (−1.2). Also, in group B, when comparing healthy samples to virus-free aphid-exposed samples, no miRNAs were differentially expressed. Among all combinations, 59 individual miRNAs (1 known and 58 novel) were upregulated, and 16 miRNAs (3 known and 13 novel) were downregulated (as determined using both statistical tools DESeq2 and edgeR) (Supplementary File S5).

### 3.4. miRNAs Expression Profiles after BYDV Infection

**Figure 5:**
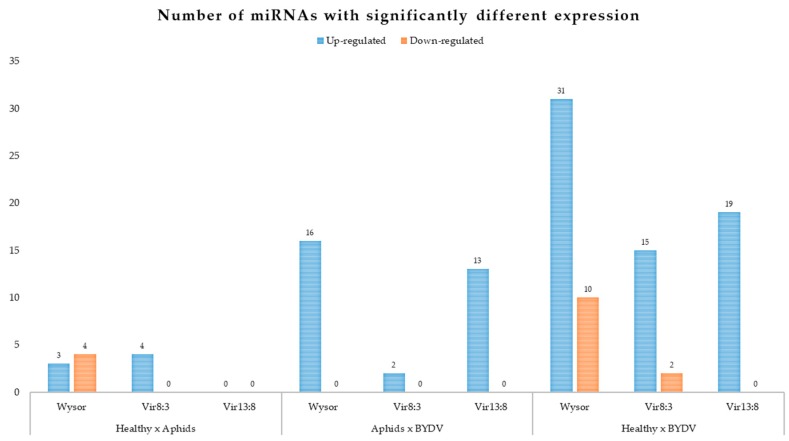
Number of differentially expressed miRNAs from barley cultivars Wysor, Vir8:3, and Vir13:8 in response to BYDV infection. The differential expression was analyzed using the R bioconductor packages DESEq2 and EdgeR. The miRNAs with a log fold-change (Log_2_ FC) more than 1.5 (1.2) or less than Log_2_FC—1.5 (1.2) and with *p*-value less than 0.05 were considered significantly differentially expressed.

The grouping of samples in all groups enabled the determination of the BYDV infection influence on the expression of individual miRNAs without the effect of the cultivar and vector. Overall, the expression of 73 miRNAs was altered (>1.5-fold) in the BYDV-infected barley leaves (Figure 5; Table 3; Supplementary File S5), while the other miRNAs were equally expressed among the treatments (healthy × aphids × BYDV). Of the 73 differentially expressed miRNAs, 58 were upregulated in response to viral infection (miRNAs), and only one (hvu-miR6188) of these was a known miRNA. Even though the hvu-miR6188 abundance was always higher in BYDV-infected samples than in the healthy ones for all barley types except Vir13:8 (average upregulation: 50 times in Vir13:8, 170 times in Wysor, over 200 times in Vir8:3), it similarly increased among treatment groups, even for the virus-free aphid control group, for a particular genotype. Likewise, the abundance of four novel miRNAs (novelMiRNA_2944, novelMiRNA_30197, novelMiRNA_4701, and novelMiRNA_7966) increased in all genotypes after interaction with BYDV-free aphids; however, the difference in expression was not significant among the barley genotypes. In Vir13:8, not a single miRNA was up- or downregulated after BYDV-free aphids fed on barley leaves in comparison to levels in the healthy control group. This very small influence of aphid sucking was somehow striking.

The most conserved pattern of upregulation as a reaction to BYDV infection was for novel miRNA10778, which did not vary in expression in any genotype in response to any treatment except BYDV infection, in which case the expression increased approximately 10–15 times. Novel MiRNA10778 belongs to the small nucleolar RNA (snor14) family (RF01280) on chromosome Chr5H. In the 15 miRNAs that tended to be downregulated after BYDV infection, two miRNAs (hvu-miR6189 and hvu-miR6203) were known miRNAs, and the others were novel. However, not one of these downregulated miRNAs had their altered level of expression confirmed among all the barley genotypes. The miRNAs with altered expression did not vary strongly in their level of expression (from 1000 to approximately 5000 reads in a sample). Furthermore, no miRNAs were expressed in only one treatment group.

### 3.5. miRNAs’ Expression Profiles in Relation to the Resistance Levels

**Figure 6:**
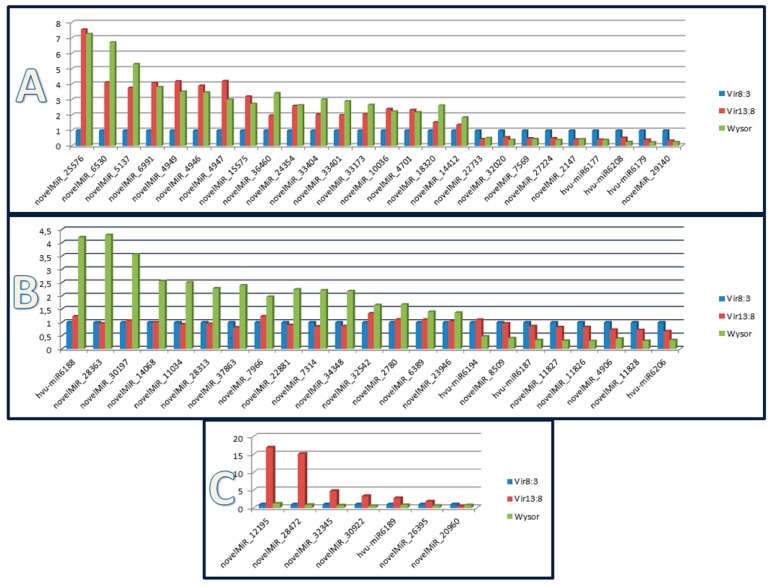
Expression of selected miRNAs as influenced by the barley genotype in response to BYDV infection. The miRNA expression in the BYDV infected group is shown. The average number of reads from the biological triplicates was used and transformed to normalized values such that a graphical comparison could be made. The values on the left axis are the fold-change values. In the top graph (**A**), miRNAs varying in their expression between Vir8:3 and the other two genotypes (Vir13:8 and Wysor) are displayed; in the middle graph (**B**), miRNAs varying in their expression between Wysor and the other two genotypes (Vir8:3 and Vir13:8) are displayed; and in the bottom graph (**C**), miRNAs varying in their expression between Vir13:8 and the other two genotypes (Vir8:3 and Wysor) are displayed.

The grouping of the samples also enabled a comparison of individual miRNAs’ expression among individual genotypes. The resistant cultivar Wysor (with *Ryd*2) was compared to the newly developed lines Vir8:3 and Vir13:8 with pyramided resistance genes. Differential expression of some miRNAs between genotypes was statistically confirmed using two-way ANOVA (*p* < 0.0005). In general, Wysor differed from Vir8:3 and Vir13:8 in the number of differently expressed miRNAs; 48 miRNAs differed in their expression level among the varieties. However, there were no miRNAs that would express uniquely in one variety and not any others. In most cases, expression differed between Wysor and both Vir8:3 or Vir13:8, or Wysor grouped with Vir13:8 in differing from Vir8:3 (Figure 6). Most of the time, the miRNAs of either Wysor, or both Wysor and Vir13:8, were upregulated compared to Vir8:3 (Figure 6). Twenty-six miRNAs differed in their expression in both Wysor and Vir13:8 in response to BYDV infection compared to their levels in Vir8:3, where most were upregulated (Figure 6A). An interesting trend was observed in the expression of some miRNAs (novelMiRNA_14412, novelMiRNA_18320, novelMiRNA_2226, novelMiRNA_4701, and novelMiRNA_4946) in that their abundance increased slightly after virus-free aphids sucked on the leaves and increased even more after BYDV infection. However, this trend varied in its intensity between genotypes; it was the lowest for Vir8:3 and most evident for resistant Wysor. The expression of the 23 other miRNAs also differed in Wysor after BYDV infection compared to lines Vir8:3 and Vir13:8 (Figure 6B); most of them were upregulated, and some were downregulated. Hvu-miR6188 also varied in its expression depending on the genotype; expression was higher in Wysor in all treatments but was most pronounced in BYDV-infected samples. Another atypically expressed miRNA was novelMiR_12195, which differed significantly in its abundance in line Vir13:8. Hundreds of copies were found in each sample in Wysor and Vir8:3 (with similar increasing trend to the miRNAs in the paragraph above), but the expression in Vir13:8 was upregulated more than 10 times for each biological triplicate (Figure 6C).

### 3.6. Target Gene Prediction for Barley miRNAs

To gain a better understanding of the regulatory roles of known and novel barley miRNAs, target genes were predicted using psRNATarget software (http://plantgrn.noble.org/psRNATarget/) by comparing miRNA sequences against the barley reference genome from Plant Genome and Systems Biology (PGSB). A total of 72 targets of 57 known miRNAs and 198 targets of 163 novel miRNA candidates were identified (Supplementary File S6). Functional annotation of these target genes showed the presence of defense-related genes (~18.71%) and transcription factors (~12.23%) that were regulated by known miRNAs. For example, the MLOC_37399.1 gene and LRR receptor-like serine/threonine-protein kinase gene were targeted by two known miRNAs (hvu-miR169 and hvu-miR6182) and 14 novel miRNAs (novelMiR10173, novelMiR_17133, novelMiR_5298, novelMiR_4859, novelMiR_18118, novelMiR_8217, novelMiR_31433, novelMiR_4949, novelMiR_15575, novelMiR_32281, novelMiR_13404, novelMiR_12515, novelMiR_8325, and novelMiR_21515) (Supplementary File S6); furthermore, 15 miRNAs targeted leucine-rich repeat receptor kinases (LRR-KRs) (AK372040). For the targets of novel miRNAs, ~19.4% were genes with unknown functions (Supplementary File S6).

### 3.7. Functional Classification of Known and Novel MiRNAs

**Figure 7:**
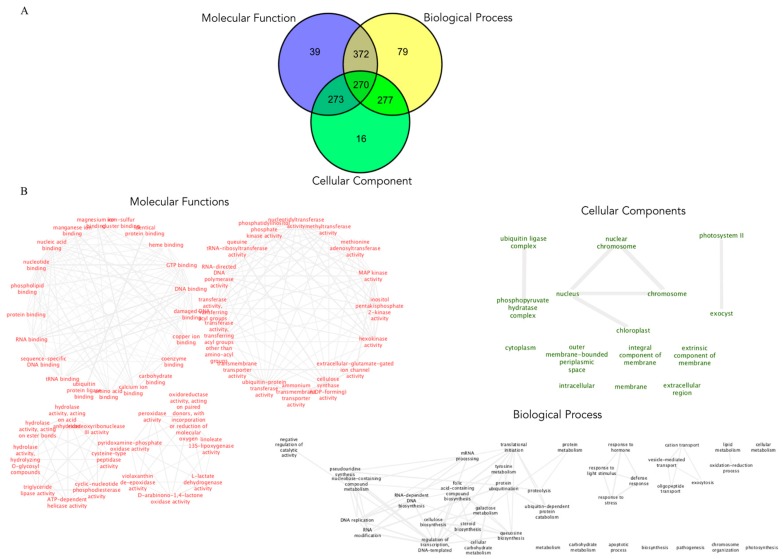
Gene ontology (GO) annotations for known and novel miRNAs from *Hordeum vulgare*: 39 for molecular function (MF), 79 for biological process (BP), and 16 for cellular component (CC). (**A**) Venn diagram for miRNAs. (**B**) Network analysis of individual GO terms: molecular function in red, biological process in black, and cellular component in green. The functional enrichment analysis of miRNAs was performed using g:GOSt implemented in the g:profile online web server (*p* < 0.05).

In the functional enrichment analysis of the 66 known and 634 novel miRNAs using g:Profile, the distribution of enriched gene ontology (GO) terms showed several noteworthy findings. For barley miRNAs, three GO terms were identified: molecular function (MF: 39 miRNAs), biological process (BP: 79), and cellular component (CC: 16) (Figure 7). Several miRNAs were associated with more than one GO term; for example, 372 miRNAs were affiliated with both MF and BP, 277 miRNAs with BP and CC, 273 miRNAs belong to CC and MF, and 270 miRNAs were associated with MF, BP, and CC (Figure 7; Supplementary File S7).

The significantly enriched MF terms included catalytic activity (GO:0003824; *p*-value = 4.16 × 10^−44^), “RNA binding” (GO:0003723; *p*-value = 1.64 × 10^−2^), small molecule binding (GO:00036094; *p*-value = 2.36 × 10^−10^), kinase activity (GO:0016301; *p*-value = 3.52 × 10^−6^), and “transporter activity (GO:0005215; *p*-value = 2.90 × 10^−3^) (Figure 7; Supplementary File S7). The significantly enriched BP terms included metabolic process (GO:0008152; *p*-value = 3.83 × 10^−110^), cellular metabolic process (GO:0044237; *p*-value = 1.66 × 10^−60^), gene expression (GO:0010467; *p*-value = 2.42 × 10^−16^), RNA biosynthesis process (GO:0032774; *p*-value = 1.37 × 10^−8^), and response to stress (GO:0006950; *p*-value = 3.91 × 10^−5^) (Figure 7; Supplementary File 7). The significantly enriched CC terms included membrane (GO:0016020; *p*-value = 5.76 × 10^−14^), cytoplasm (GO:0005737; *p*-value = 6.46 × 10^−4^), and nucleus (GO:0005634; *p*-value = 2.05 × 10^−2^) (Figure 7; Supplementary File S7).

### 3.8. Functional Domain Architecture Analysis of miRNAs Targets

Functional analysis of proteins targeted by known and novel miRNAs by classifying them into families and predicting domains and important sites was performed using InterPro, as described above. Depending upon the functional module present, targeted proteins had a single domain (SD) or multiple domains (MD). For example, the serine/threonine-protein phosphatase protein targeted by hvu-miR169, hvu-miR6182, novelMiR_10173, novelMiR_17133, novelMiR_5298, novelMiR_4859, novelMiR_18118, and novelMiR_8217 possessed a SD, such as metallophosphoesterase domain (InterPro ID: IPR004843). MD proteins targeted by miRNAs, including LRR-kinase, possessed leucine-rich repeat (InterPro ID: IPR001611) and leucine-rich repeat-containing N-terminal, type 2 domains (InterPro ID: IPR013210). Receptor kinase 2 proteins targeted by novelMiR_27343, novelMiR_1471, novelMiR_6062, novelMiR_10950, and novelMiR_6539 possessed leucine-rich repeat (InterPro ID: IPR001611) and protein kinase-like domains (InterPro ID: IPR011009) (Supplementary File S6).

### 3.9. RT-qPCR Validation of the Expression of 20 miRNAs

The miRNAs (17 novel miRNAs and 3 known miRNAs) that differed in their expression between the control and BYDV groups were validated (Supplementary File S1). Correlation analyses revealed Pearson correlation values between RT-qPCR and sRNA-seq of individual miRNAs ranging between 0.263 (novelMiR_36460) and 0.987 (novelMiR_2944) with an average *R* = 0.774 (Supplementary File S8), suggesting that the sequencing data was in general consistent with the RT-qPCR results.

### 3.10. siRNA Analysis in Barley Cultivars

**Figure 8:**
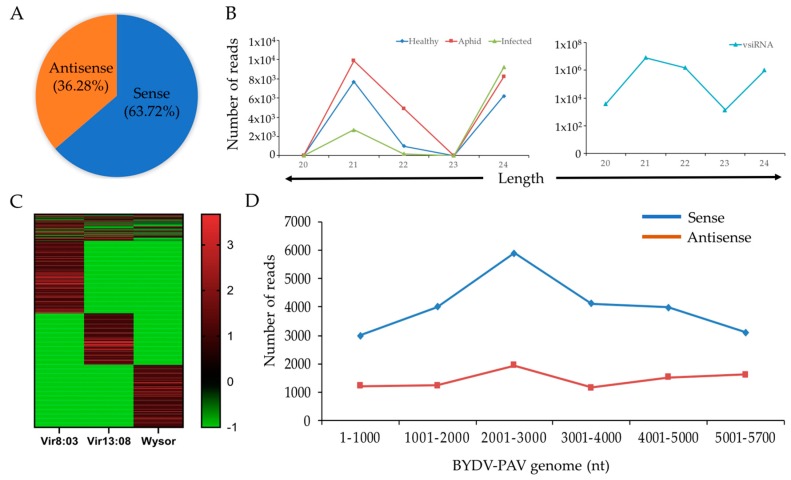
siRNA analysis in healthy, aphid-infested, and Barley yellow dwarf virus (BYDV)-infected barley genotypes. (**A**) Distribution of sense and antisense vsRNAs calculated as a percentage. (**B**) Length distribution of siRNAs derived from plants (upper) and the BYDV-PAV strain of Barley yellow dwarf virus (lower). X-axis: length of vsRNAs (nucleotides). Y-axis: number of reads. (**C**) Abundancy of BYDV-derived siRNAs in BYDV-infected samples of individual genotypes. Values are scaled logarithmically (value 0 is graphed as −1). (**D**) distribution of vsRNAs on the BYDV-PAV genome. X-axis: BYDV genomic position. Y-axis: number of reads.

The origin of siRNA in healthy, aphid-infested, and BYDV-infected Barley cultivars viz. Vir8:3, Vir13:8 and Wysor was determined (see Section 2.7). Overall, 85.39% of siRNA reads were found to be host-specific, i.e., specific to *Hordeum vulgare*, while 14.61% were derived from BYDV-PAV. The length distribution of plant-derived siRNAs showed the abundance of 21 nt, followed by 24 nt and 22 nt (Figure 8B). The size distribution of BYDV-PAV-specific siRNAs (virus-derived small RNAs (vsRNAs)) showed the higher accumulation of 21 nt, followed by 22 nt and 24 nt (Figure 8B). There was a slight predominance of sense vsRNAs (64%) compared to antisense vsRNAs (36%) (Figure 8A). Higher frequencies of matches occurred in the RNA-dependent RNA polymerase (RdRp) area of the BYDV genome, which was true mainly for the sense-orientated vsRNAs (Figure 8D).

The abundance of siRNAs among individual genotypes in BYDV-infected samples was compared using two-way ANOVA (Figure 8C). Most of the siRNAs were uniquely distributed in one genotype only. The majority of siRNAs significantly differed in their abundance between the genotypes. Altogether, 755 siRNAs reads covering the BYDV-PAV genome were identified (Supplementary File S9).

## 4. Discussion

In the past two decades, extensive studies revealed that, apart from their role in plant development, miRNAs also orchestrate plant innate immunity, leading to antiviral immunity or viral pathogenesis [36]. In barley, roles have been identified for miRNAs for plant development [37,38] and responses to abiotic stresses, such as drought [39,40,41,42,43], heat [44], phosphorus deficiency [45,46], and boron [47]. To our knowledge, our study is the first to identify miRNAs that change in response to a biotic stress. We found more than 600 novel miRNAs in barley and examined their level of expression after BYDV infection in genotypes of barley that differ in their resistance to BYDV. The novel miRNAs’ expression patterns were confirmed using RT-qPCR.

In our comparison of the miRNA responses in the two new multi-resistance lines Vir13:8 and Vir8:3 with the *Ryd*2-carrier Wysor to BYDV infection, genotypes moderately influenced the expression of the individual miRNAs. In a comparison of the expression of individual miRNAs, slight differences in the three genotypes were found. Notably, Wysor had the most profound reaction to BYDV infection in the number of miRNAs that changed in the expression after infection and in the intensity of those changes. Vir13:8 had just a few less miRNAs that changed, whereas Vir8:3 differed most from the other two genetic materials. However, the profile of the miRNAs expressed was not affected by the genotype; there were no miRNAs expressed specifically in just one genotype. Although most miRNAs are highly conserved across various species [12], different miRNA profiles have been recorded among different plant varieties [48,49]. We used varieties with a similar genetic background, as opposed to usual studies in which sensitive and resistant varieties were used. Here, we aimed at confirming similar patterns instead of looking for differences. Thus, our results are not surprising.

Profiling of siRNAs showed a typical predominance of 21- and 22-nt vsRNAs that have been previously reported by many other studies [50,51,52,53]. There was a prevalence of sense vsRNAs, which is in accordance with other studies [54]. There were no significant differences in abundance of vsRNAs among different genotypes. On the other hand, the BYDV siRNAs analysis showed a very specific distribution of individual siRNAs among the genotypes. This is in accordance with results of other studies [55,56], even though these studies compared susceptible and resistant varieties. In one study, no correlation was found between the virus titer and the vsRNAs abundance in samples of tobacco and tomato [57]. Without any further investigations, no conclusion can be drawn regarding the barley genotypes resistance level based on vsiRNAs analyses.

Furthermore, none of the miRNAs were unique for any of the treatment replicates (healthy, aphid-infected, and BYDV-infected). In maize, two miRNAs were present only in virus-infected samples [52], and in grapevine, six were unique to virus-infected plants [49]. In *Arabidopsis*, differences in miRNAs profiles were also found after virus infection [58]. In a study by Du et al. [20], the miRNA profile varied in rice plants that were infected by rice stripe virus (RSV) compared with those infected with rice dwarf virus (RDV). The virus species can therefore also influence the miRNA profile and expression. Another aspect that can shed more light on the influence of a virus species influence is the abundance and length distribution of the miRNAs. Many studies have consistently recorded that miRNA levels generally increase in plants infected with viruses [49,58,59,60,61]. However, most of these studies included two closely related viruses such as TMV and oilseed rape mosaic virus (ORMV), or undefined mixed infections [49]. In other studies, the level of miRNAs was not affected by the presence of a virus [52], or the level changed in response to only one virus but not another [20]. The same applies to the length distribution of the miRNAs [20,52,58]. The miRNA expression profile and pattern should therefore be regarded as specific to given conditions. Also, bear in mind that changes in miRNA levels in virus-infected cells do not necessarily lead to corresponding changes in the transcriptome [58].

The miRNA that responded to BYDV infection most significantly was novelMiR_10778, which was specifically upregulated in the presence of the BYDV virus, regardless of the barley genotype (Supplementary File S3). NovelMiR_10778 belonged to the family of snoR14 (Rf01280) (Supplementary File S4). Small nucleolar RNAs (snoRNAs) are a class of small RNA molecules that primarily guide chemical modifications of other RNAs, mainly ribosomal RNAs, transfer RNAs, and small nuclear RNAs. The H/ACA box snoRNAs (to which snoR14 belongs) are associated with pseudouridylation. H/ACA snoRNAs are structurally characterized by the presence of a double hairpin, each harboring a pseudouridylation pocket, which is specific to a particular target sequence based on base pairing [62]. Furthermore, another significantly responding miRNA was novelMIR_22504, with a possible target gene of tRNA pseudouridine synthase. Recently, pseudouridylation has been recognized as a regulator of viral latency processes in human immunodeficiency virus (HIV) infections [63].

According to target prediction analysis (Supplementary File S6), novelMiR_10778 is involved in the regulation of the gene for the succinyltransferase component of 2-oxoglutarate dehydrogenase complex (OGDHC). OGDHC is a multienzyme system comprising three catalytic components: OGDH (2-oxoglutarate dehydrogenase) (E1o, EC1.2.4.2), dihydrolipoyl succinyl transferase (E2o, EC 2.3.1.61), and dihydrolipoyl dehydrogenase (E3, EC 1.8.1.4) [64]. OGDHC catalyzes a highly regulated step of the mitochondrial tricarboxylic acid cycle and is essential for development and survival [65]. In potato (*Solanum tuberosum*), OGDHC has been shown to be limiting for respiration and plays an important role in nitrogen assimilation [66]. Mammals have natural mechanisms for upregulating OGDHC in response to stress [65]. Further studies into the role of novelMiR_10778 in BYDV infection in barley might therefore reveal similar functions.

A further miRNA that reacted to BYDV infection was novelMiR_1724 with a predicted gene target being the receptor-like protein kinase. The expression of this gene has been previously demonstrated to be influenced by virus infection [52,67].

A very small number of miRNAs were up- or downregulated as a result of aphid infestation. No miRNAs changed in expression specifically in response to aphid infestation in the three genotypes of barley. When the expression of a specific miRNA changed, the change was usually followed by a similar, even greater change in the BYDV-infected plants, which indicates a general biotic stress response. In a study by Sattar et al. [68], melon lines had different patterns of conserved and newly identified miRNA expression profiles during different stages of aphid herbivory. Also, Xia et al. [69] found that different miRNAs were expressed in chrysanthemum during aphid feeding, with the greatest differences during early stages of aphid infestations (2–6 h of infestation). Here, we sampled 14 days after the beginning of infestation as our aim was not to study the effect of the insect, but that of the virus; the aphid-infested plants served only as a control to filter out the effect of the insect stress on the plants.

## 5. Conclusions

We identified 746 miRNAs in barley. The expression of 73 miRNAs changed significantly after the genotypes were infected with BYDV. The conserved upregulation of novel miRNA10778 was particularly strong across barley genotypes. In addition, 48 miRNAs differed in their expression among the genotypes. The RT-qPCR results confirmed the sequencing results. The miRNA expression profile in response to BYDV was similar between the newly developed pyramided lines Vir8:3 and Vir13:8 compared to the *Ryd*2-carrying Wysor. This study provides insight into the expression patterns of miRNAs in response to BYDV in barley and should help in further studies aiming at developing novel strategies for crops against virus infection.

## Figures and Tables

**Table 1:** Length distribution across individual biological triplicates. Total reads of all miRNAs were calculated.

Length	Reads								
	Vir08:3-Healthy	Vir08:3-Aphids	Vir08:3-BYDV	Vir13:8-Healthy	Vir13:8-Aphids	Vir13:8-BYDV	Wysor-Healthy	Wysor-Aphids	Wysor-BYDV
17 nt	0	0	0	0	0	0	0	0	0
18 nt	682,998	682,560	745,280	811,167	645,012	452,149	416,998	532,958	331,169
19 nt	331,899	306,980	264,569	185,988	187,166	158,464	236,887	281,412	164,734
20 nt	222,129	223,179	246,226	228,309	264,705	204,136	199,589	198,959	154,057
21 nt	133,407	151,047	173,220	182,222	238,100	152,760	127,623	128,664	127,267
22 nt	175,074	202,756	191,915	183,252	207,757	164,975	188,409	172,958	194,110
23 nt	647,606	642,026	521,699	743,495	545,439	553,835	616,627	459,322	510,693
24 nt	3103	2950	3041	3563	5634	2891	3685	3420	3277
25 nt	0	0	0	0	0	0	0	0	0
Total	2,196,216	2,211,498	2,145,950	2,337,996	2,093,813	1,689,210	1,789,818	1,777,693	1,485,307

**Table 2:** Most abundant miRNA families in three barley genotypes under BYDV infection and healthy controls.

No. of Novel miRNAs	No. of Annotated Novel miRNAs	No. of RNA Families
680	662	161
**Ten most abundant RNA families**		
RNA family ID	No. of novel miRNA	RNA type
RF00906	159	MIR1122
RF00100	33	7SK
RF00028	31	Intron_gpl
RF00001	29	5S_rRNA
RF00230	27	T-box
RF01766	18	cspA
RF01417	18	RSV_RNA
RF00026	17	U6
RF01959	16	SSU_rRNA_archaea

**Table 3:** miRNAs with the greatest changes in expression after BYDV infection.

Name of miRNA	No. of Strikes	miRNA Sequence	Accession ID **	Annotation
Novel miRNA10778	5	gaggtctctgtagatgatga	AK364815	Dihydrolipoyllysine-residue succinyltransferase component of 2-oxoglutarate dehydrogenase complex
Novel miRNA144	5	aaggtccctgacgtctggtac	AK363491	Fructose-1,6-bisphosphatase class 1
Novel miRNA1724	5	ccattatagaatgatgctggcgt	AK361660	Receptor-like protein kinase
Novel miRNA2226	5	ttgccgagagctgctcagat	MLOC_44527.2	Unknown protein
Novel miRNA22504	5	ctttgtcatagttactctgatag	AK357280	tRNA pseudouridine synthase family protein
Novel miRNA2944	5	gtttatagtggaatctctaaaag	MLOC_61720.1	S-adenosylmethionine synthase
Novel miRNA36460	4	ggatagagcatggagaacgta	AK364238	Ent-copalyl diphosphate synthase
Novel miRNA4701	4	tttaaaaccggtttgtatcaca		
Novel miRNA7557	4	aattttatcgcgatcgga	AK376576	Potassium transporter 10
Novel miRNA7558	4	aattttatcgcgatcgga	AK376576	Potassium transporter 10
Novel miRNA14068	4	gacgactagatagcgacaggctc	MLOC_72356.1	Lachrymatory factor synthase
Novel miRNA18320	4	agtcttcgcgtctggatggacga	MLOC_69330.1	Retrotransposon protein, putative, Ty1-copia subclass
Novel miRNA24228	4	tttaaaaccggtttgtatcaca		
Novel miRNA30197	4	atccaacggtgcggacgcgcggg	AK375972	L-lactate dehydrogenase
Hvu-miR6188 *	4	gguggaucgaugaacccggcga	MLOC_67894.2	Aquaporin

Number of strikes signifies the number of groups (A to E) in which the expression of given miRNA was significantly higher for BYDV infected samples as compared to the healthy samples. The miRNAs in bold are those whose expression was also significantly higher in the majority of lines/varieties, when aphid samples were compared to the BYDV samples (this way the false effect of aphid sucking was excluded). *: Hvu-miR6188 is explained in the text in a separate paragraph in Section 3.5. **: PGSB accession number.

## Supplementary Materials

The following are available online at https://www.mdpi.com/2223-7747/9/1/60/s1.
